# Should clinical laboratories adapt to the reality of chronic kidney disease in the determination of parathyroid hormone?

**DOI:** 10.1515/almed-2021-0046

**Published:** 2021-06-21

**Authors:** María Luisa González-Casaus, Pilar Fernández-Calle, Antonio Buño Soto

**Affiliations:** Department of Laboratory Medicine, Hospital Central de la Defensa Gomez Ulla, Madrid, Spain; Department of Laboratory Medicine, Hospital Universitario La Paz, Madrid, Spain

**Keywords:** analytical variability, biological variability, chronic kidney disease (CKD), intact parathyroid hormone (PTH)

## Abstract

**Objectives:**

The contribution of the clinical laboratory to diagnostics is increasingly important since a great deal of clinical decisions rely on laboratory test results.

**Content:**

Parathyroid hormone (PTH) measurement presents a considerable analytical variability due to the heterogeneity of its circulating forms and the antigenic configuration of the different assays commercially available. Such variability may have an impact on pathological conditions associated with significant increases in circulating PTH, as it is the case of chronic kidney disease (CKD).

**Summary:**

Despite the recent identification of new molecules involved in bone and mineral disorders associated with CKD, such as klotho or the fibroblastic factor 23 (FGF23), nephrologists still base their clinical decisions on PTH concentrations. The problem is that unawareness of these analytical considerations may cause errors in the clinical interpretation of test results.

**Outlook:**

This systematic review addresses these issues from the clinical laboratory perspective and proposes new approaches related to PTH method selection and result expression. These new strategies will help laboratory medicine specialists and nephrologist better determine the status of CKD patients.

## Introduction

The role of the clinical laboratory in the diagnosis and follow-up of numerous diseases is increasingly relevant, since clinical decisions largely rely on laboratory test results. Therefore, a deep understanding of each analyte is not only crucial for the appropriate interpretation of results, but also for selecting the most adequate assay that will provide the most relevant information. In this regard, parathyroid hormone (PTH) determination, an essential tool to evaluate mineral homeostasis, is a clear example.

The main problem of PTH determination in serum or plasma is the variability of results. Unawareness of this problem may result in interpretation errors and inadequate clinical decisions. As it occurs in many other analytes, PTH variability is influenced by preanalytical factors inherent to the biology of the analyte and sample handling. Added to preanalytical issues, other analytical factors lead to an important inter-assay variability of PTH inducing to diagnostic errors, especially in those diseases associated with excessive increases in circulating PTH levels, as it is the case of chronic kidney disease (CKD). Analytical variability does not only hinder interlaboratory comparison, but it also makes it necessary to consider “tendencies” rather than absolute values.

## Factors contributing to preanalytical PTH variability

PTH determination is conditioned by significant biological variability (ethnicity, age, and body mass index [BMI]), is influenced by the circadian rhythm and is extremely sensitive to calcemia changes, since extracellular ionized calcium is the main regulator of PTH secretion and synthesis. In some seconds, hypocalcemia induces PTH secretion through the activation of a calcium-sensing receptor [1] in the membrane of parathyroid cells, whereas hypercalcemia rapidly inhibits the release of PTH and enhances its degradation. Thus, assessing calcium levels in blood is critical to the correct interpretation of PTH results.

Apart from ionized calcium, other factors are involved in parathyroid function, including phosphorus [2], calcitriol (1,25-dihydroxyvitamin D) [3], and fibroblast growth factor 23 (FGF23) [4]. PTH regulates calcium homeostasis by increasing bone resorption and tubular calcium reabsorption, and increasing vitamin D synthesis in the kidney. Hence, it is important to take into account that the very prevalent hypovitaminosis D status in the general population influences PTH results, as it causes secondary hyperparathyroidism. Even more, the threshold value of serum calcidiol (25-hydroxyvitamin D, reserve marker) below which PTH secretion increases to maintain calcitriol values (active vitamin D) within physiological ranges is considered a biochemical criterion for defining vitamin D deficiency. According to this, the lower values of serum calcidiol registered in the black population could explain their higher PTH circulating levels, as compared to the white population.

Additionally, the use of biotin supplementation may interfere with test results, depending on the immunoassay employed [5]. In any case, it is important to take into account that the biological matrix used for PTH determination and sample storage conditions influence PTH results. PTH is more stable in plasma than in serum and when stored at 4 °C rather than at room temperature. The Workgroup on PTH of the International Federation of Clinical Chemistry (IFCC) [6] recommends that in ethylenediaminetetraacetic acid (EDTA) samples, the plasma be separated within 24 h after venous puncture and analyzed within 72 h when stored at 4 °C. Serum can be the sample of choice if analyzed within 3–4 h after collection or if stored at −20 °C, since it has the advantage of concurrent calcium determination. Evidence has been provided [7] that thrombin in rapid serum tubes (RST tubes) leads to 14% decreases in PTH results, as compared to standard serum separator tubes (SST). This may be explained by changes in PTH antigenicity induced by the action of thrombin. Finally, the sampling site also influences PTH results, since PTH concentrations in blood drawn from a central venous catheter are 30% higher than those determined in peripheral blood [6]. This is important as, although peripheral blood sampling is more frequent, central venous catheter sampling is sometimes used in special situations, as in patients on hemodialysis or candidates to parathyroid surgery. The sampling site should be detailed in laboratory analysis requests to ensure the correct interpretation of results, especially in serial PTH measurements.

## Analytical variability of PTH

In addition to these preanalytical factors described above, PTH particularly presents a remarkable analytical variability, with important differences depending on the measurement method used [8]. This is generally overlooked, as suppliers provide very similar reference intervals. A range of factors explain the significant interassay variability observed in PTH determination. Of special mention is the heterogeneity of its circulating peptides, followed by differences in the antigenic configuration (which means that not all assays detect the same circulating forms) and origin of the calibrators used by manufacturers to standardize their immunoassays.

### Heterogeneity of circulating PTH peptides

**Figure 1:**
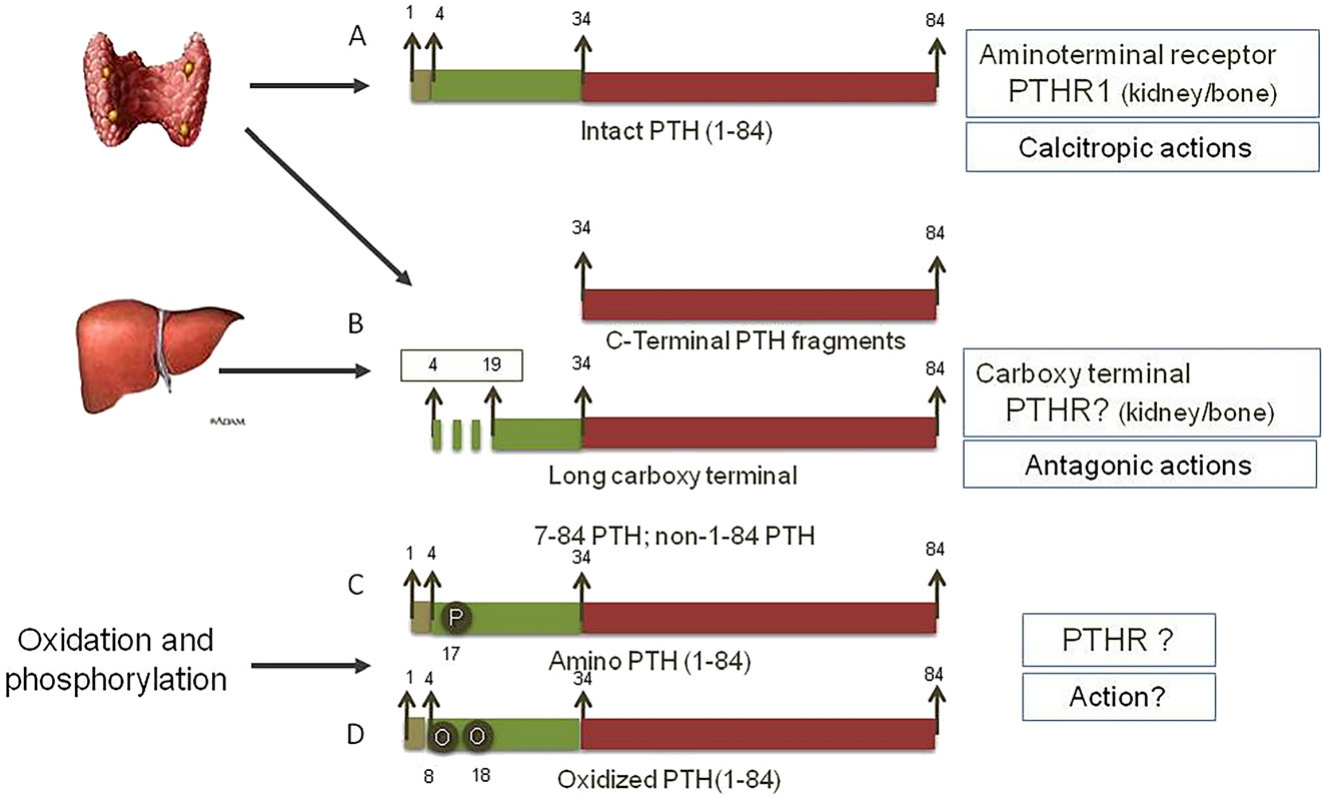
Circulating PTH peptides. Circulating PTH peptides is a combination of: [A] Biologically active intact 1–84 PTH, released in response to hypocalcemia to increase circulating levels of calcium through the activation of PTH receptor-1 (PTHR1); [B] Carboxy-terminal peptides resulting from intraglandular and hepatic degradation of full-length 1–84 PTH, of which around 10% are large peptides broken down between the amino acid segment 4–19 (Amino-terminal truncated PTH, 7–84 PTH or non 1–84 PTH) and with antagonistic effect to 1–84 PTH through the activation of a carboxy-terminal PTH receptor (unidentified); [C] Amino-PTH, phosphorylated 1–84 PTH with unknown action and [D] PTH oxidized at amino acid 8 and 18, which are post-translational processes that interfere in the binding to PTHR1.

Circulating PTH (Figure 1) is a combination of (a) *intact 1–84 PTH*, biologically active, released by the parathyroid gland in response to hypocalcemia to increase circulating calcium levels through the aminoterminal activation of PTH receptor-1 (PTHR1) in the kidneys and the bones; and (b) a variety of *carboxy-terminal peptides*, a product of intraparathyroid and hepatic degradation of full-length 1–84 PTH. By 10% of these PTH peptides are large C-terminal fragments split in the aminoacid region 4–19, called amino-terminal truncated PTH, 7–84 PTH, or non 1–84 PTH [9]. Of note, these fragments are not inactive, but they exert an antagonistic effect on 1–84 PTH through the activation of a carboxy terminal PTH receptor (not yet been identified) [10]. To further complicate these circulating forms, PTH undergoes post-translational oxidation processes in methionine residues in amino acids 8 and 18 [11] and phosphorylation processes, especially in the serine residues of amino acid 17, known as amino-PTH, which is overexpressed in diseases such as parathyroidal carcinoma [12]. These two modifications limit the ability of 1–84 PTH to activate PTHR1.

### Differences between PTH assay methods

**Figure 2:**
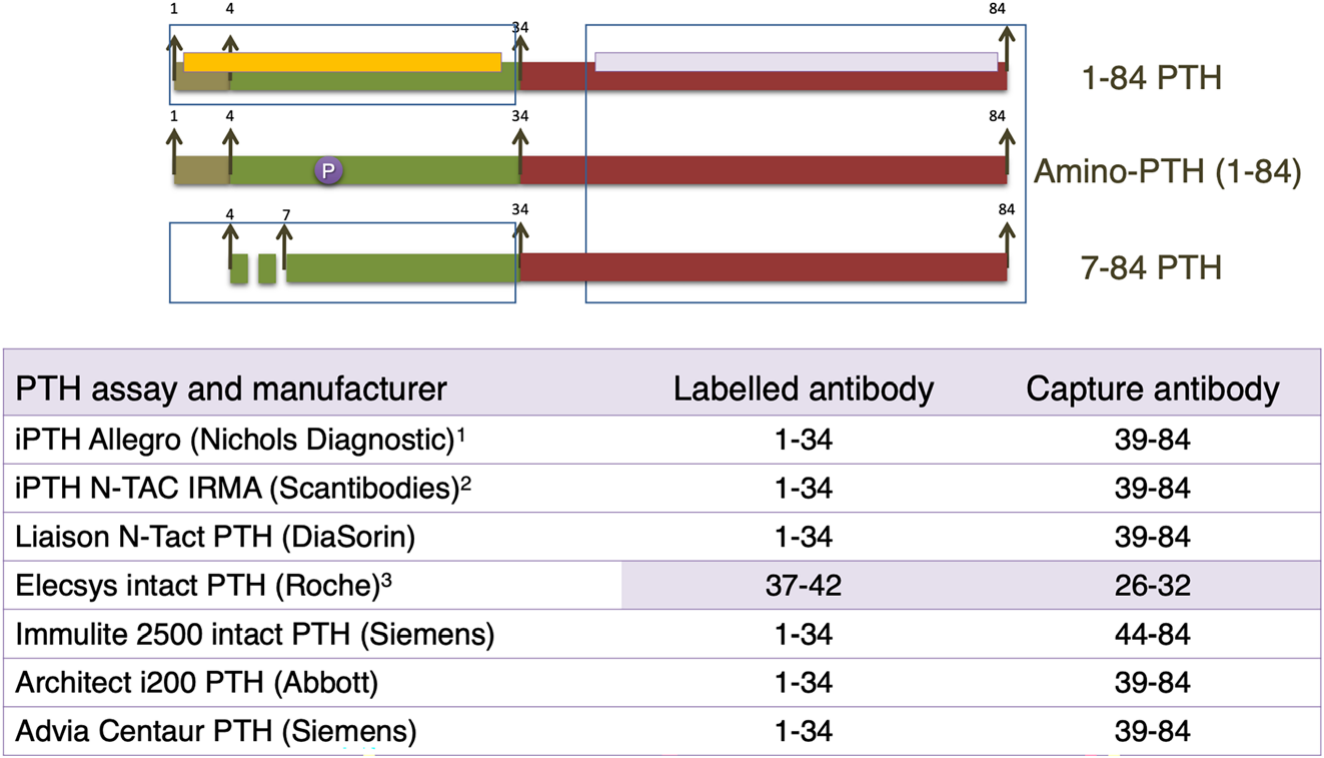
Second-generation assays. Since in the past they were thought to exclusively measure biologically active 1–84 PTH (7–84 PTH had not yet been identified), second generation assays are incorrectly known as “intact PTH assays”, but they have cross reactivity with large C-terminal fragments 7–84. As these large fragments, with antagonistic actions to 1–84 PTH, are cleared by the kidneys, their/its percentage increases as glomerular filtration decreases renal. This condition must be taken into account when intact PTH is measured in CKD patients. (1) This assay, no longer used, was validated by bone histomorphometry and was the assay of reference in most nephrology guidelines. (2) Isotopic IRMA assay. (3) Intact PTH assay with a different configuration than the other automated CLIAs. It shows interference with amino-PTH, since its amino-terminal antibody (curiously, the capture antibody) is very distal.

**Figure 3:**
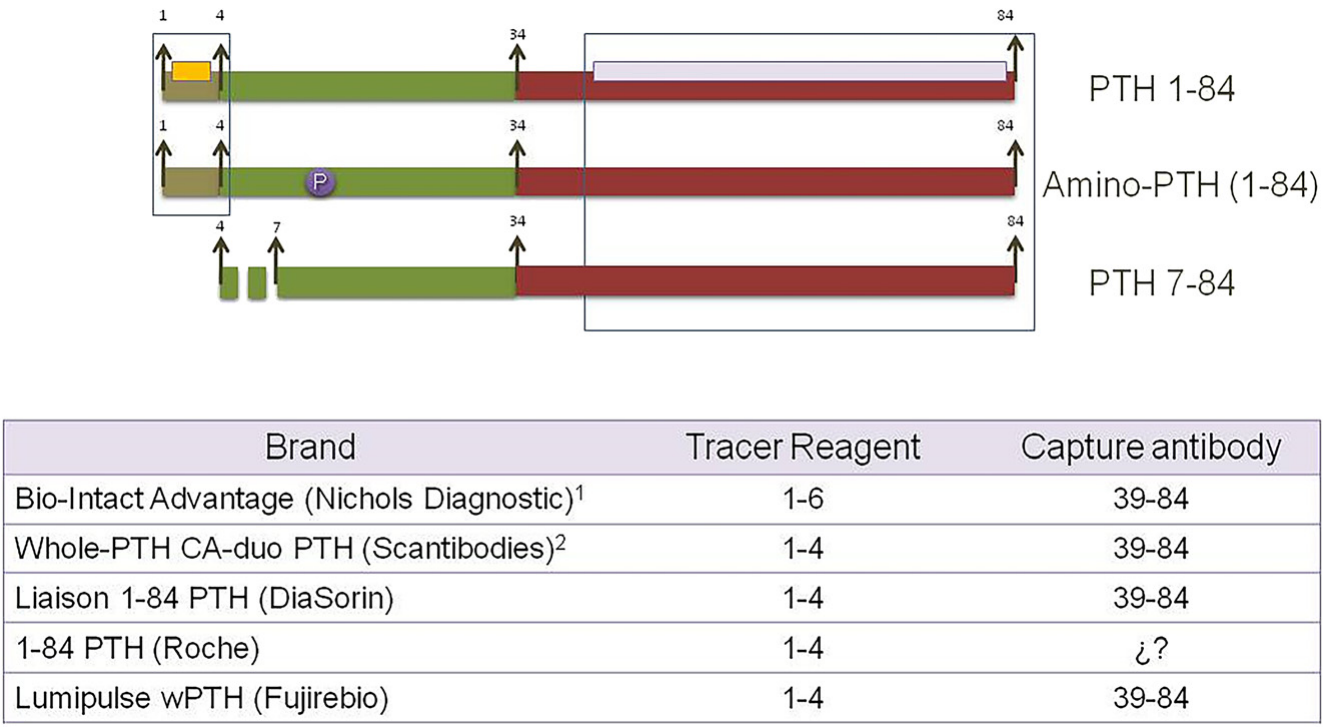
Third-generation assays. Called bio intact PTH assays, they are developed to avoid 7–84 PTH interference and the consequent determination of antagonistic effects against PTH. To such purpose, its tracer only recognizes the 4–6 first aminoacids of the aminoterminal end. (1) It is no longer available in the market due to severe precision problems that led the manufacturer to bankruptcy. (2) Non automated isotopic IRMA assay.

The second cause of interassay variability is the antigenic configuration of the different methods for PTH measurement. *First generation* methods, developed in 1980, were competitive radioimmunoassays (RIA) with low sensitivity and specificity, with a single antibody generally targeted to the carboxy terminal or mid PTH regions. Thus, these assays measured both, 1–84 PTH and all its circulating forms. In 1987 the *second generation of PTH methods* appeared (Figure 2) [13, 14]; these assays used two antibodies, a first carboxy terminal that immobilizes the molecule, and a second one targeted to the amino terminal region, which is labelled and allows PTH quantitation. These are known as “intact PTH assays” (i-PTH) since, until 7–84 PTH was identified [9], they were thought to only measure active 1–84 PTH. This inaccurate term is still employed, although antagonistic effects of the hormone are measured as a result of their interference with 7–84 PTH. To avoid such interference, *third generation* assays were developed, known as biointact PTH (bio-PTH) o PTH-whole (Figure 3). These assays do not measure 7–84 PTH because the second antibody only recognized the first 4–5 amino acids of the molecule [15]; however, they present interference with PTH forms that have undergone post translational modifications.

Taking into account these differences in the antigenic configuration of assays, discrepancies between second- and third-generation assays are expected as long as they quantify different circulating peptides. Surprisingly, there are also significant inconsistencies between the assays of the same generation [16], with a −40 and 120% range of deviation with respect to the IRMA i-PTH used as reference in the assessment of the first clinical practice guidelines of the *International Kidney Foundation* [17]. This alarming situation is due to the fact that these i-PTH assays were calibrated by manufacturers using different standards from distinct origins (bovine, murine…) since at that time, the international recombinant human 1–84 PTH standard had not yet been established. This situation results in a lack of commutability [8], [16].

## Impact of the analytical variability of PTH on kidney disease

The problems associated with i-PTH assay methods raised concerns about potential errors in the interpretation of results, especially in the context of CKD, where PTH determination is essential for the assessment of mineral metabolism impairment. The loss of nephrons and renal klotho expression, which triggers an adaptative increase in fibroblastic growth factor 23 (FGF23) resulting in vitamin D inhibition and hypocalcemia, promotes the development of secondary hyperparathyroidism associated with bone anomalies. These bone disorders are globally termed ‘renal osteodystrophy’ and involve both, low and high bone turnover diseases. PTH is the choice test for noninvasive diagnosis of renal osteodystrophy, since the gold-standard method, which is bone biopsy and hystomorphometric study after double labeling with tetracyclines, is not feasible in clinical practice. Even more, although earlier and more sensitivity biomarkers of impaired mineral homeostasis in CKD, such as FGF23 and klotho, have been recently identified, clinical guidelines [17], [18] still base therapeutic decisions on circulating PTH levels. The problem posed by i-PTH immunoassays, especially in CKD patients, is that the proportion of 7–84 PTH increases as glomerular filtrate (GF) declines, because of their kidney clearance, accounting for 50% of circulating forms of PTH at stage 5 CKD. This limitation does not affect other diseases such as primary hyperparathyroidism (secondary to hyperplasia, adenoma or carcinoma, of one or several parathyroid glands) or other causes of secondary hyperparathyroidism (i.e. after bariatric surgery or in a context of hypovitaminosis D), with lower increases in PTH serum levels and normal kidney function, where the percentage of 7–84 PTH is not significant.

**Figure 4:**
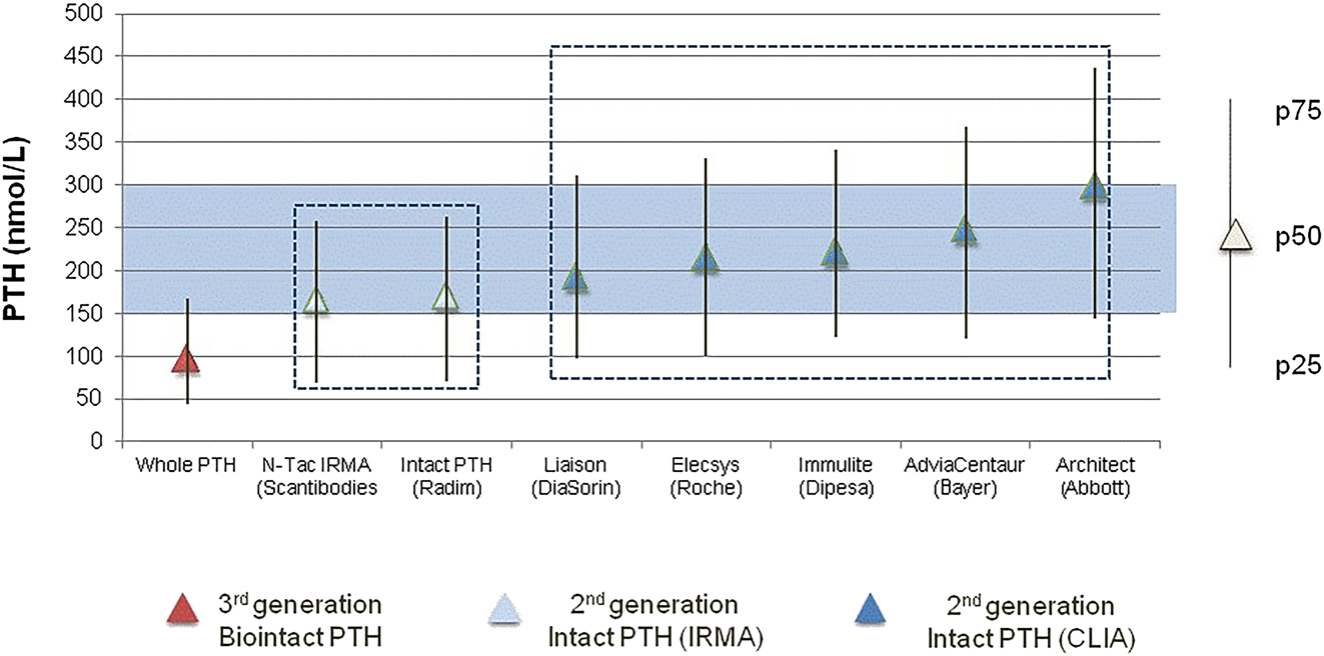
Differences in the percentile distribution of a population of stage-5 CKD patients on hemodialysis across the different PTH assays (SEN* study). As expected, the results obtained with the biointact PTH assay are lower than the ones obtained with intact PTH assays, as they do not determine 7–84 PTH. However, important differences are also observed between second-generation assays, with isotropic IRMA assays yielding lower values than automated CLIA assays. These differences are surprising, especially, discrepancies in the median values and percentiles obtained with the Architect assay (Abbott). *Data from the SEN study [18].

**Figure 5:**
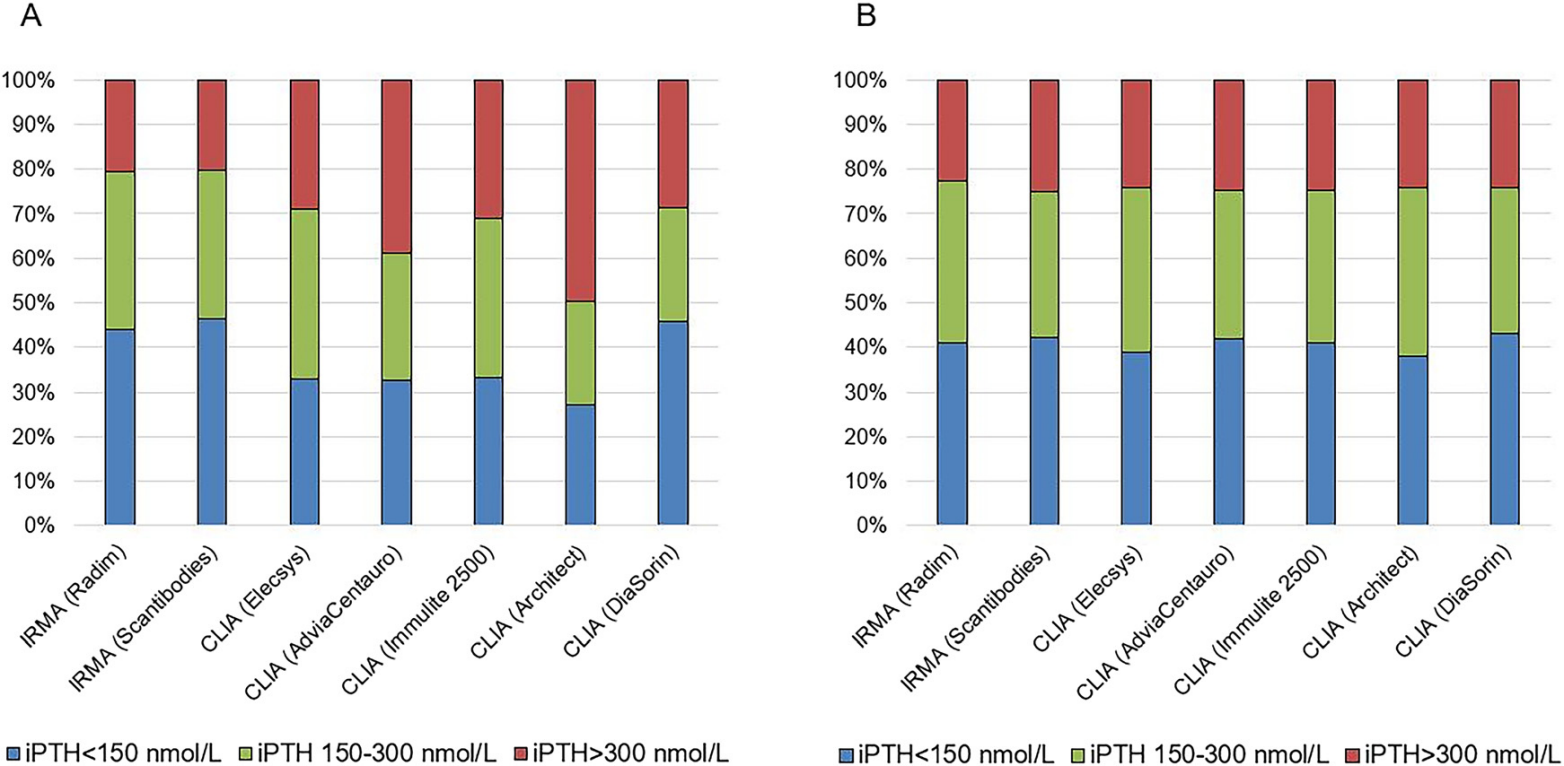
Differences in the categorization of KDOQI stage-5 CKD patients on hemodialysis as a function of the iPTH assay employed (SEN study*). KDOQI guidelines recommend a PTH interval of 150–300 nmol/L for patients with stage-5 CKD on dialysis based on the correlation between the isotropic iPTH “Allegro” assay (Nichols Institute) and bone histomorphometry. Circulating levels of iPTH <150 nmol/L suggest low-bone remodeling, whereas values >300 nmol/L indicate high-bone remodeling. [A] When patients are stratified according to the "raw" iPTH values obtained, significant differences are observed in the percentages of each category, especially in CLIA Architect (Abbott), which shows that half of the patients would be identified as with high-bone remodeling and would be candidates to treatment with anti-parathyroid hormones. [B] When the corresponding adjustment algorithms for hemodialysis of the SEN study are applied [18], only 25% of patients would require a treatment for hyperparathyroidism and poor clinical decisions would have been made. *Data from the SEN study [18].

The identification of interassay variability in i-PTH measurements and its implication in the clinical management of CKD patients challenged the validity of clinical guidelines such as the “*National Kidney Foundation/Kidney Dialysis Outcomes Quality Initiative*” (NKF/KDOQI) [17], which recommended specific i-PTH ranges for each CKD stage, based on the correlation of a second-generation assay (iPTH IRMA Allegro*; Nichols Institute Diagnostics Inc*) with histomorphometry in bone biopsies. Indeed, subsequent guidelines “*Kidney Disease-Improving Global Outcomes*” (KDIGO) [18] consider these limitations and recommend the use of broader ranges (2–9 times above the upper limit of normality (UNL) for each assay). The Spanish Society of Nephrology (SEN) designed a series of studies to identify potential equivalences between the most widely used i-PTH immunoassays in Spain and in relation with the only bio PTH method available at this moment [19]. Higher values were obtained by automated chemiluminescent i-PTH assays (CLIA), as compared to immunoradiometric assays (IRMA). Of note, significant differences were observed between CLIA assays, which affected clinical decision (Figures 4 and 5). Fortunately, the excellent correlation among all the immunoassays studied allowed developing a set of interassay adjustment algorithms and a result equivalence cards were designed to facilitate the implementation of KDOQI guidelines in patients with stage 5 CKD [19]. It is important to note that the first SEN algorithms were established in a CKD stage 5 population prevalent on hemodialysis (HD), when the proportion of circulating 7–84 PTH is maximal. This means that these interassay equivalence algorithms were not applicable to earlier CKD stages, where the proportion of carboxyterminal fragments is lower [20]. Even more, differences in the percentage of circulating forms of PTH also were observed depending on the type of dialysis. For an equal i-PTH value, patients on peritoneal dialysis (PD) show a lower proportion of 1–84 PTH than HD patients, as consequence of higher levels of circulating ionized calcium due to loss of proteins through the peritoneum [21]. This situation explains the higher prevalence of adynamic bone disease in patients on PD vs HD and it is important to aware that the use of the first SEN adjustment algorithms (assessed in HD population) in PD patients, instead of the specific ones for PD [22], could further iatrogenically “adinamize” these patients. In any case, the solution to PTH inter-assay variability, which mainly affects CKD, should not be the use of adjustment algorithms, but the claim for adequate standardization.

## Position/perspective of clinical laboratories

In this scenario, third-generation PTH assays emerge as a promising alternative in the management of CKD patients. However, the only third-generation assay available has long been the dual IRMA assay (CA-PTH Duo Scantibodies), which included an i-PTH assay and a bio-PTH assay [23], but its isotopic character, the lack of automatization and its elevated cost, hindered the widespread use of this method by clinical laboratories. In the past, an automated chemiluminescence bio PTH assay (CLIA) developed by Nichols Institute Diagnostics Inc (Nichols Advantage Bio-Intact PTH) was implemented in numerous laboratories, but important precision problems reported with shifts of +29 and +52% [24], between years 2003 and 2005, leads to the bankrupt of the manufacturer and, the disappearance of i-PTH Allegro, (used as reference in KDOQI guideline) a year later. This situation forced the return to i-PTH methods, which are currently used in the majority of clinical laboratories, even knowing that they quantify antagonistic effects of the hormone and recognizing their limitations. Indeed, the KDIGO guidelines still recommend the use of second-generation assays for their availability in all clinical laboratories.

### Biointact PTH methods in renal osteodystrophy

Fortunately, novel bio PTH assays offer us the opportunity to solve this “step back” [25]. Their implementation at clinical level gets us closer to the reality of the biologically active molecule. Indeed, occasionally, the combination of i-PTH and bio PTH also provide additional data for the assessment of renal osteodystrophy. Such is the case of the diagnosis of low bone remodeling, where higher discrepancies occur between PTH and bone hystomorphometry [26]. To minimize potential misdiagnosis of bone remodeling, some authors suggest other alternatives to report PTH results, such as the 1–84/7–84 PTH ratio [27] or even the combination of this ratio with i-PTH (i-PTH values <420 pg/mL associated to a 1–84/7–84 PTH < 1 ratio in CKD 5D patients would be predictive of low bone remodeling, with a 90% sensitivity) [28]. A limitation of these ratios is to assume that 7–84 PTH concentration equals the difference between i-PTH and bio PTH. However, this assumption is not appropriate since the oxidized and phosphorylated forms of circulating PTH, as amino-PTH, have cross-reactivity with bio PTH methods but they are not detected by most i-PTH immunoassays (whose antibodies identify the amino terminal segment that includes the phosphorylated amino acid). Nevertheless, it could be useful for the diagnosis and management of those diseases where amino-PTH is overexpressed [12]. Under physiological conditions, the lower proportion of amino-PTH than large 7–84 PTH fragments results in a bio PTH/i-PTH ratio [(1–84 *PTH + aminoPTH*)*/*(1–84 *PTH + 7–84 PTH*)] less than 1 (except for the Roche i-PTH assay that detects 1–84 PTH + 7–84 PTH + amino-PTH, as its epitopes recognize the 26–32 and 37–42 aminoacid region of PTH). The inversion of this ratio would/could identify a possible parathyroidal carcinoma among those patients with primary hyperparathyroidism [29, 30]. Similarly, the reversal of this ratio due to amino-PTH overexpression would reflect a severe parathyroid hyperplasia in the context of secondary hyperparathyroidism in, setting/assessing the indication of treatment with Cinacalcet (a calcimimetic drug that corrects secondary hyperparathyroidism by increasing the calcium-sensing receptor´s sensitivity to calcium in parathyroid gland) or a parathyroidectomy [31].

Added to the potential clinical use of these ratios, the implementation/use of bio PTH methods would allow a better standardization and comparison of CKD patients against the healthy population of reference without the interference of 7-84-PTH, whose concentration varies significantly depending on the glomerular filtrate or type of dialysis. This would reduce biological uncertainty to its measurement, despite cross-reactivity with oxidized PTH and amino-PTH. We should not forget that oxidized PTH increases in CKD patients by oxidative stress, and the oxidized methionines interfere with PTH/PTHR1 interaction, resulting in a loss of activity. The recent development of a method for measuring oxidated PTH will help understand its physiopathological role and clinical implications [11], [32].

### Standardization of biointact PTH methods

Additionally, the clinical standardization of third-generation methods offers the opportunity of correcting and avoiding unnecessary and uncertain interassay adjustments by requiring the calibration of all PTH immunoassays commercially available against a universal *gold standard* reference. In October 2009, the WHO Workgroup on International Biological Standards established the first international human recombinant standard for 1-84-PTH (WHO International Standard; NIBSC code: 95/646). Therefore, some groups [33] started to refer their automated bio PTH assays (Roche Elecsys y DiaSorin Liaison), previously calibrated against other standards to that human recombinant one. As result of this restandardization, potential interassay differences are eliminated and new biological reference intervals can be established. In line with this criterion, manufacturers as Fujirebio subsequently developed and calibrated their automated bio PTH method against the NIBSC code 95/646 standard. Sensitive to this problem, the IFCC created an expert workgroup on PTH to improve the comparability of PTH results [6]. Among the aims of this workgroup, it is precisely to demand/claim calibration of all PTH assays against this international standard and to assess the reference method for PTH measurement. At present, the combination of liquid chromatography/mass spectrometry (LCM/MS) is the current candidate, still pending optimization [34].

In relation to quality specifications, the first Strategic Conference of the European Federation of Clinical Chemistry and Laboratory Medicine (EFLM), held in Milan in 2014 reached a consensus on the validity of the hierarchical model of Stockholm. This model was simplified into three models: Standards based on clinical impact, biological variation, and state-of-the-art. In view of the scarcity of evidence on the clinical impact of analytical variability on clinical decision-making, biological variability emerges as the approach better focused on the clinical use of PTH. In addition, the EFLM Workgroup on biological variation (BV), along with the workgroup for the development of a BV database, designed the critical appraisal checklist (BIVAC) [35]. Based on this standard, this task group is conducting a European multicentric experimental study called EUBIVAs to assess BV in multiple analytes, including bio PTH [36], with within-subject variation coefficients (CV_I_) of 14.7 (95% CI: 14.0–15.5), whereas the estimated CV_I_ derived from the meta-analysis conducted by this workgroup on PTH was 24.2 (IC 95% 20.2–25.9) (https://biologicalvariation.eu/bv_specifications/measurand#).

## Possible future adaptation of the PTH measurement in clinical laboratories to approach the reality of CKD population

Despite these considerations, third-generation immunoassays have not yet been introduced into clinical practice for the management of CKD patients. The main problem that hinders their implementation in clinical practice is the limited experience and lack of guidelines for this type of assays (for clinicians, what is not recommended in guidelines does not exist, even although it is beneficial). Its standardization vs osteodystrophy is challenging, if we consider that biopsy bone histomorphometry is an invasive, high-cost and less feasible method. Some studies associate the isotopic bio PTH assay (Scantibodies laboratories Inc.) with bone biopsies, such as the Herberth study about ratios [28] and even more, indirect data are available from its comparison and adjustment with the i-PTH “Allegro” used in KDOQI guidelines [8]. According to this study, the KDOQI recommended 150–300 pg/mL interval of i-PTH [17] in CKD stage 5 is 84–165 pg/mL of Scantibodies bio PTH [8]. In any case, the adaptation of bio PTH to KDOQI recommendations for early CKD stages is not challenging since guidelines recommend i-PTH values as in healthy population. More difficult is to define bio PTH values for CKD stage 4 for which guidelines establish a range of iPTH of 70–110 pg/mL. However, despite the contribution of oxidized PTH, this small increase in i-PTH levels results from the accumulation of long carboxy-terminal fragments due to the glomerular filtrate rate reduction and therefore, bio PTH should be maintained in ranges near to those of earlier stages, as it lacks cross-reactivity with these fragments. However, we cannot forget that the prevalence of hypovitaminosis D is even higher in CKD patients than in the general population, which will expectedly have a significant impact on circulating 1–84 PTH.

Standardized PTH thresholds obtained using third-generation assays should be established for the CKD population. Although KDOQI guidelines, which were initially intended for noninvasive diagnosis of bone involvement in CKD, are still in use, recent advances in the understanding of mineral metabolism disorders associated with CKD actually places us in a different scenario. In 2006, the KDIGO working group [37] changes the classic concept of kidney osteodystrophy (a term that specifically remains restricted to CKD-associated bone disorders) and replaces it by the term CKD/MBD (Chronic Kidney Disease- Mineral and Bone Disorders) to emphasize both the early occurrence of these disorders (from the first CKD minute) and the involvement of extraskeletal tissues, especially cardiovascular tissue, which is the main cause of mortality in CKD patients. Hence, the new paradigm in the management of these disorders goes beyond the hyperparathyroidism and renal osteodystrophy correction, by pursuing other benefits, especially to delay the disease progression and preventing cardiovascular mortality. From this point of view, and considering the unfeasibility of bone biopsies, the reference ranges for bio PTH should not be established/assessed through the distribution and comparison of results in patients with different CKD stages from healthy population, even more if we are evaluating adaptive mechanisms. The clinical evaluation of bio-PTH should be based on robust survival studies assessing mortality and progression to dialysis. A study was recently conducted to assess circulating levels of 1–84 PTH (DiaSorin) associated with these adverse effects in 1812 CKD patients with an estimated glomerular filtrate (eFG) between 15 and 45 mL/min [38] and a mean follow-up of 52 months. Most patients exhibit PTH values above the upper limit of normality (ULN: 39.4 pg/mL), which doubles in patients with eFG below 20 mL/min. The reported risk threshold based on cardiovascular events was 42.9 pg/mL for eFG ≥ 30 mL/min; 104.6 pg/mL for eFG 20–29 mL/min; and 134 pg/mL for eFG <20 mL/min; whereas the risk thresholds based on progression to dialysis were 53.5 pg/mL for eFG ≥ 30 mL/min; 49.4 pg/mL for eFG 20–29 mL/min and 93.7 pg/mL for eFG <20 mL/min.

Further studies are needed to improve our understanding of PTH and its circulating forms. From our perspective, it is time to start to use third-generation assays, to evaluate their clinical application, and demonstrate that their theoretical advantage over i-PTH assays really facilitates the monitoring of parathyroid function and clinical decision-making in CKD. Recent advances in the understanding of the etiopathogenic mechanisms of mineral homeostasis associated with CKD also must find their response in/from the clinical laboratory. Measurement of both bio PTH and FGF23 [39] in blood should be a basic diagnostic tool in the management of CKD/MBD. As laboratory medicine specialists, we should promote protocol initiatives in collaboration with other clinical specialists with the aim to improve the quality of our results. Now or never!

## References

[1] Brown EM, Gamba G, Riccardi D, Lombardi M, Butters R, Kifor O (1993). Cloning and characterization of an extracellular Ca(2+) sensing receptor from bovine parathyroid. Nature.

[2] Almaden Y, Hernandez A, Torregrosa V, Canalejo A, Sabate L, Fernandez-Cruz L (1998). High phosphate levels directly stimulate parathyroid hormone secretion and synthesis by human parathyroid tissue in vitro. J Am Soc Nephrol.

[3] Silver J, Naveh-Many T, Mayer H, Schmeizer HJ, Popovtzer MM (1986). Regulation by vitamin D metabolites of parathyroid hormone gene transcription in vivo in the rat. J Clin Invest.

[4] Ben-Dov IZ, Galitzer H, Lavi-Moshayoff V, Goetz R, Kuro -OM, Mohammadi M (2007). The parathyroid is a target organ for FGF23 in rats. J Clin Invest.

[5] Piketty ML, Polak M, Flechtner I, Gonzales-Briceño L, Souberbielle JC (2017). False biochemical diagnosis of hyperthyroidism in streptavidin-biotin-based im- munoassays: the problem of biotin intake and related interferences. Clin Chem Lab Med.

[6] Sturgeon CM, Sprague S, Almond A, Cavalier E, Fraser WD, Algeciras-Schimnich A (2017). IFCC Working Group for PTH. Perspective and priorities for improvement of parathyroid hormone (PTH) measurement: a view from the IFCC Working Group for PTH. Clin Chem Acta.

[7] La´ulu SL, Straseski JA, Schmidt RL, Genzen JR (2014). Thrombin-mediated degradation of parathyroid hormone in serum tubes. Clin Chim Acta.

[8] Souberbielle JC, Boutten A, Carlier MC, Chevenne D, Coumaros G, Lawson-Body E (2006). Inter-method variability in PTH measurement: implication for the care of CKD patients. Kidney Int.

[9] Brossard JH, Cloutier M, Roy L, Lepage R, Gascon-Barre M, D´Amour P (1996). Accumulation of a non-(1-84) molecular form of parathyroid hormone (PTH) detected by intact PTH assay in renal failure: importance in the interpretation of PTH values. J Clin Endocrinol Metab.

[10] Murray TM, Rao LG, Divieti P, Bringhurst FR (2005). Parathyroid hormone secretion and action: evidence for discrete receptors for the carboxyl-terminal region and related biological actions of carboxyl- terminal ligands. Endocr Rev.

[11] Ursem SR, Vervloet MG, de Jongh RT, Heijboer AC (2020). Oxidation of parathyroid hormone. Clin Chim Acta.

[12] Rubin MR, Silverberg SJ, D’Amour P, Brossard JH, Rousseau L, Sliney J (2007). An N-terminal molecular form of parathyroid hormone (PTH) distinct from hPTH(1 84) is overproduced in parathyroid carcinoma. Clin Chem.

[13] Nussbaum SR, Zahradnik RJ, Lavigne JR, Brennan GL, Nozawa-Ung K, Kim LY (1987). Highly sensitive two-site immunoradiometric assay of parathyrin, and its clinical utility in evaluating patients with hypercalcemia. Clin Chem.

[14] Blind E, Schmidt-Gayk H, Armbruster FP, Stadler A (1987). Measurement of intact human parathyrin by an extracting two-site immunoradiometric assay. Clin Chem.

[15] Gao P, Scheibel S, DÀmour P, John MR, Rao SD, Schmidt-Gayk H (2001). Development of a novel immunoradiometric assay exclusively for biological active parathyroid hormone 1-84: implication for improvement of accurate assessment of parathyroid function. J Bone Miner Res.

[16] Ureña Torres P (2006). The need for reliable serum parathyroid hormone measurements. Kidney Int.

[17] National Kidney Foundation (2003). K/DOQI clinical practice guidelines for bone metabolism and disease in chronic kidney disease. Bone metabolism and disease in chronic kidney disease. Am J Kidney Dis.

[18] Eckardt K-U, Kasiske BL (2009). Kidney Disease Improving Global outcomes (KDIGO) clinical practice guideline for the diagnosis, evaluation, prevention and treatment of chronic kidney disease-mineral and bone disorder (CKD-MBD). Kidney Int.

[19] de la Piedra C, Fernández E, González Casaus ML, González Parra E (2008). Diferencias en la función de los péptidos paratiroideos. ¿Qué estamos midiendo?. Nefrología.

[20] Herberth J, Fahrleitner-Pammer A, Obermayer-Pietsch B, Krisper P, Holzer H, Malluche H (2006). Changes in total parathyroid hormone (PTH, PTH-(1-84) and large C-PTH fragments in different stages of chronic kidney disease. Clin Nephrol.

[21] Sanchez-Gonzalez C, Gonzalez-Casaus ML, Lorenzo V, Albalate M, Torregrosa JV, Mas S (2018). Higher proportion of non 1-84 fragments in peritoneal dialysis patients compared to hemodialysis patients using solutions containing 1,75 mmol/L calcium. Front Physol.

[22] González Casaus ML, González Parra E, Sanchez Gonzalez C, Albalate M, de La Piedra C, Fernandez E (2014). La menor proporción de parathormona circulante biológicamente activa en diálisis peritoneal no permite el ajuste intermétodo de parathormona establecida para hemodiálisis. Nefrología.

[23] John MR, Goodman WG, Gao P, Cantor TL, Salusky IB, Juppner H (1999). A novel immunorradiometric assay detects full-length human PTH but not amino-terminally truncated fragments: implications for PTH measurements in renal failure. J Clin Endocrinol Metab.

[24] Cantor T (2005). Parathyroid hormone assay drift: unappreciated problem in dialysis patient Management. Semin Dial.

[25] Hecking M, Kainz A, Bielesz B, Plischke M, Beilhack G, Hörl WH (2012). Clinical evaluation of two novel biointact PTH (1-84) assays in hemodialysis patients. Clin Biochem.

[26] Barreto FC, Barreto DV, Moysés RM, Neves KR, Canziani ME, Draibe SA (2008). K/DOQI-recommended intact PTH levels do not prevent low-turnover bone disease in hemodialysis patients. Kidney Int.

[27] Monier-Faugere MC, Geng ZP, Maward H, Friedler RM, Gao P, Cantor TL (2001). Improved assessment of bone turnover by the PTH (1-84) large C-PTH fragments ratio in ESRD patients. Kidney Int.

[28] Herberth J, Branscum AJ, Mawad H, Cantor T, Monier-Faugere MC, Malluche HH (2010). Intact PTH combined with the PTH ratio for diagnosis of bone turnover in dialysis patients: a diagnostic test study. Am J Kidney Dis.

[29] Caron P, Maiza JP, Renaud C, Cormier C, Barres BH, Soubervielle JC (2009). High third generation/second generation PTH ratio in a patient with parathyroid carcinoma: clinical utility of third generation/second generation PTH ratio in patients with primary hyperparathyroidism. Clin Endocrinol.

[30] Cavalier E, Daly AF, Betea D, Pureanu-Apretii PN, Delanaye P, Stubbs P (2010). The ratio of parathyroid hormone (PTH) measured by third- and second-generation assays as a marker of parathyroid carcinoma. J Clin Endocrinol Metab.

[31] Cavalier E, Betea D, Schleck ML, Gadisseur R, Vroonen L, Delanaye P (2014). The third/second generation assay ratio as a marker for parathyroid carcinoma: evaluation using an automated platform. J Clin Endocrinol Metab.

[32] Hocher B, Oberthur D, Slowinski T, Querfeld U, Schaefer F, Doyon A (2013). Modeling of oxidized PTH (oxPTH) and non-oxidized PTH (n- oxPTH) receptor binding and relationship of oxidized to non-oxidized PTH in children with chronic renal failure, adult patients on hemodialysis and kidney transplant recipients. Kidney Blood Press Res.

[33] Cavalier E, Delanaye P, Lukas P, Carlisi A, Gadisseur R, Souberbielle JC (2014). Standardization of DiaSorin and Roche automated third generation PTH assays with an International Standard: impact on clinical populations. Clin Chem Lab Med.

[34] Kumar V, Barnidge DR, Chen LS, Twentyman JM, Cradic KW, Grebe SK (2010). Quantification of serum 1-84 parathyroid hormone in patients with hyperparathyroidism by immunocapture in situ digestion liquid chromatography-Tandem mass spectrometry. Clin Chem.

[35] Aarsand AK, Røraas T, Fernandez-Calle P, Ricos C, Díaz-Garzón J, Jonker N (2018). The biological variation data critical appraisal checklist: a standard for evaluating studies on biological variation. Clin Chem.

[36] Bottani M, Banfi G, Guerra E, Locatelli M, Aarsand AK, Coskun A (2020). European Biological Variation Study (EuBIVAS): within-and between-subject biological variation estimates for serum biointact parathyroid hormone based on weekly samplings from 91 healthy participants. Ann Transl Med.

[37] Moe S, Drüeke T, Cunningham J, Goodman W, Martin K, Olgaard K (2006). Definition, evaluation and classification of renal osteodystrophy: a position statement from Kidney Disease: improving Global Outcomes (KDIGO). Kidney Int.

[38] Canney M, Djurdjev O, Zierold C, Block F, Wolf M, Levin A (2019). GFR-specific versus GFR-agnostic cutoffs for parathyroid hormone and fibroblast growth factor 23 in advanced chronic kidney disease. Am J Nephrol.

[39] Gonzalez-Casaus ML, Gonzalez-Parra E, Fernandez-Calle P, Buño Soto A (2020). FGF23: De la nefrología de salón a la cabecera del paciente. Nefrología.

